# Incidence, management, and outcome of incidental meningioma: what has happened in 10 years?

**DOI:** 10.1007/s11060-023-04482-5

**Published:** 2023-11-08

**Authors:** Olivia Näslund, Per Sveino Strand, Ole Solheim, Mohammad Al Masri, Okizeva Rapi, Erik Thurin, Asgeir S. Jakola

**Affiliations:** 1 Department of Clinical Neuroscience, Institute of Neuroscience and Physiology, Sahlgrenska Academy, University of Gothenburg, Gothenburg, Sweden; 2 Department of Radiology, Sahlgrenska University Hospital, Gothenburg, Sweden; 3 Department of Neurosurgery, Sahlgrenska University Hospital, Gothenburg, Sweden; 4 Department of Neurosurgery, St. Olavs Hospital, Trondheim University Hospital, Trondheim, Norway; 5 Department of Neuromedicine and Movement Science, Norwegian University of Science and Technology, Trondheim, Norway; 6 Sahlgrenska Academy, University of Gothenburg, Gothenburg, Sweden; 7 Department of Surgery, Sahlgrenska University Hospital, Östra, Gothenburg, Sweden; 8 Institute of Neuroscience and Physiology, Sahlgrenska Academy, Blå stråket 7, 41345 Gothenburg, Sweden

**Keywords:** Meningioma, Incidental, Asymptomatic, Management, Outcome

## Abstract

**Purpose:**

The aim of this study was to study the use of brain scanning, and the subsequent findings of presumed incidental meningioma in two time periods, and to study differences in follow-up, treatment, and outcome.

**Methods:**

Records of all performed CT and MRI of the brain during two time periods were retrospectively reviewed in search of patients with presumed incidental meningioma. These patients were further analyzed using medical health records, with the purpose to study clinical handling and outcome during a 3 year follow up.

**Results:**

An identical number of unique patients underwent brain imaging during the two time periods (n = 22 259 vs. 22 013). In 2018–2019, 25% more incidental meningiomas were diagnosed compared to 2008–2009 (n = 161 vs. 129, *p* = 0.052). MRI was used more often in 2018–2019 (26.1 vs. 12.4%, *p = 0.004*), and the use of contrast enhancement, irrespective of modality, also increased (26.8 vs. 12.2%, *p < 0.001*). In the most recent cohort, patients were older (median 79 years vs. 73 years, *p = 0.03*). Indications showed a significant increase of cancer without known metastases among scanned patients. 29.5 and 35.4% of patients in the cohorts were deceased 3 years after diagnosis for causes unrelated to their meningioma.

**Conclusions:**

Despite the same number of unique patients undergoing brain scans in the time periods, there was a trend towards more patients diagnosed with an incidental asymptomatic meningioma in the more recent years. This difference may be attributed to more contrast enhanced scans and more scans among the elderly but needs to be further studied. Patients in the cohort from 2018 to 2019 more often had non-metastatic cancer, with their cause of scan screening for metastases. There was no significant difference in management decision at diagnosis, but within 3 years of follow up significantly more patients in the latter cohort had been re-scanned. Almost a third of all patients were deceased within 3 years after diagnosis, due to causes other than their meningioma.

**Supplementary Information:**

The online version contains supplementary material available at 10.1007/s11060-023-04482-5.

## Introduction

Meningiomas account for 39% of all primary tumors of the central nervous system, and 62% of meningiomas are diagnosed radiologically [1]. An incidental meningioma is defined as an asymptomatic meningioma detected unexpectedly or due to neuroimaging for unrelated or unspecific symptoms [2]. Several studies have reported a prevalence of meningioma close to 1% in the general population [3–8], and with increasing age, the prevalence has been shown to increase, with a prevalence of 1.7% among 80-year-olds [9]. Due to the advances and widespread use of neuroimaging, incidental meningiomas are becoming more common [10–13], and more incidental than symptomatic meningiomas are now being diagnosed [14].

Recently, a substantial increase in the detection of smaller, incidental meningiomas has been shown by the Surveillance, Epidemiology and End Result database, reporting that between 2004 and 2012 the proportion of diagnosed meningiomas < 1 cm in diameter increased from 6 to 11% per year [15]. Further, nearly doubling of overall incidence rates has from 1998 to 2002 compared to 2014–2018 was seen [1]. With increasing incidence, it becomes more evident that there are many challenges unique to the management of incidental meningioma in comparison to their symptomatic counterparts. However, there are only few guidelines for follow-up and management of incidental meningioma, based on weak evidence and expertise.

In the present population-based study we sought to assess the incidence of presumed incidental meningioma in a single center, being a regional referral center for brain tumor surgery, between the years 2008–2009 compared to 2018–2019. The aim was to study the incidence of meningioma in our population, clarify changes in clinical handling over time, and potential differences in management and outcome.

## Materials and methods

### Patients

Sahlgrenska University hospital is the sole provider of brain tumor surgery in the region of Western Sweden serving a population of approximately 1.7 million. Records of performed CT (computer tomography) and MRI (magnetic resonance imaging) of the brain at the radiological departments of each of the affiliated hospital sites between the years of 2008 to 2009, and 2018 to 2019, were retrospectively reviewed. The time periods were chosen so to have a 3 year follow up in both groups. Also records of performed CT and MRI in community hospitals within our region, as well as in the private sector, were obtained.

From these records the number of individual patients undergoing CT or MRI examinations were extracted and using the keyword “meningioma”, with a variety of imaginable spellings, a list of patients with possible meningiomas was extracted and screened. Some of the extracted data formed a part of the ongoing multicenter study IMPACT and was further expanded on for the purpose of this study [16, 17]. The radiological report and electronic health journals of these patients were reviewed for the possibility of their tumor being a first time diagnosed incidental meningioma at the time of examination. An incidental meningioma was defined as a tumor with suspected origin from the meninges, with no symptoms at time of diagnosis, or symptoms that could not be attributed to the tumor. Patients with previously known incidental meningioma, or if the meningioma was not deemed incidental (that is; symptomatic), where excluded from further analysis.

Subsequently, data was extracted from the hospital medical records, including age, sex, previous medical history, WHO (World Health Organization) performance status, and reasons for brain imaging leading up to diagnosis, as well as presence of deficits at the time of diagnosis. The number of meningiomas found in each patient was recorded. Axial major and minor axis, and coronal/sagittal major axis was measured in millimeter (mm). Tumor location was recorded, and the location classified as either convexity, parasagittal, parafalcine, sphenoid wing, anterior midline, posterior fossa (midline or lateral/posterior), tentorial, intraventricular, or pineal region. Further management decisions were noted, and classified as active monitoring, active treatment with either surgery, stereotactic radiosurgery or fractionated radiotherapy, discharge from outpatient care, lost to follow-up or death. In the case of active monitoring, the above-mentioned radiological characteristics were recorded at each monitoring scan, as well as the following management decision. The presence of new meningioma-related symptoms was recorded at each follow-up. Overall outcome classified as “deceased” or “alive” was noted (before end of follow up January 31st, 2022), with date of death and cause of death recorded if applicable.

### Statistical analyses

Statistical analyses were performed using IBM SPSS version 29 software. A *p* value of < 0.05 was considered statistically significant. All tests were two-sided, and central tendencies were presented as means ± SD or median and interquartile range if skewed. Normality was assessed using Kolmogorov–Smirnov test. Continuous data was analyzed using independent sample *t* test or Mann-Whitney *U* test as appropriate. Similarly, categorical variables were analyzed using Pearson’s Chi-square or Fishers exact test.

## Results

### Patient selection

Between 2008 and 2009 31,166 brain CT or MRIs were carried out in 22,259 patients, and 129 patients with incidental meningioma were identified. Between 2018 and 2019 35,941 brain CT or MRIs were carried out in 21,987 patients, and 161 patients with incidental meningioma were identified (Supplementary Fig. 1). The number of meningiomas found in 2018–2019 was not significantly higher (*p* = 0.052). This results in a detection rate of incidental meningioma of 0.6% for patient scanned in the first period and 0.7% in the most recent period. The first diagnostic scan was a CT in 15,923 patients scanned from 2008 to 2009 and in 14,356 patients between 2018 and 2019, meaning that among the patients with a diagnosed incidental meningioma, 71.5% vs. 65.2% were suspected/diagnosed from an initial CT (*p* < 0.001). In the period 2008–2009 contrast enhancement was used in 1942 (12.2%) of all diagnostic scans, irrespective of modality, and in 3842 (26.8%) scans during 2018–2019 (*p* < 0.001). Among patients consequently diagnosed with an incidental meningioma, MRI was the diagnostic modality in 12.4% in 2008–2009, and 26.1% in 2018–2019 (*p* = 0.004). This gives CT a detection rate of 0.7% in 2008–2009, and 0.8% in 2018–2019. The detection rate with MRI as the diagnostic modality was 0.3% in 2008–2009, and 0.6% in 2018–2019.

The indication for scan causing diagnosis of incidental meningioma was not significantly different in individuals under 70 years of age (*p* = 0.5). However, among individuals over 70 years of age indication for scan was significantly different between the two time periods (*p* = 0.046). Suspected cerebrovascular incident and head injury were overall the two most common indications (Table 1). In both time periods, suspected cerebrovascular incidents and cognitive symptoms were more common among patients over 70 years of age (32.9% vs. 5.7%, resp. 19.8% vs. 11.1%), whilst headache was more common cause for imaging in those under 70 years (13.2% vs. 3.9%, resp. 9% vs. 1.7%). In 2008–2009, regardless of age-group, it was more common to be scanned due to vertigo (13.6% and 13.1%) as compared to 2018–2019 (4.4% and 6%). Among younger individuals, scans to rule out brain metastases was almost equally as common in 2008–2009 and 2018–2019 (7.5 vs. 9%). However, among elderly individuals diagnosed with an incidental meningioma, no patients were scanned to search for brain metastases from 2008 to 2009, but from 2018 to 2019 this was the indication for the diagnostic scan in 10.3% of patients diagnosed with meningioma 70 years or older.

**Table 1:** Indication for first diagnostic scan

Indication for scan*	2008–2009		2018–2019	
	Age < 70**	Age ≥ 70***	Age < 70**	Age ≥70***
Headache, n (%)	7 (13.2)	3 (3.9)	4 (9)	2 (1.7)
Cerebrovascular incident, n (%)	3 (5.7)	25 (32.9)	5 (11.1)	23 (19.8)
Head injury, n (%)	13 (24.5)	14 (18.4)	10 (22.2)	30 (26)
Audiovestibular symptoms, n (%)	1 (1.9)	1 (1.3)	0 (0)	1 (0.9)
Visual symptoms, n (%)	1 (1.9)	2 (2.6)	5 (11.1)	2 (1.7)
Psychiatric symptoms, n (%)	3 (5.7)	2 (2.6)	1 (2.2)	3 (2.6)
Cognitive symptoms, n (%)	0 (0)	9 (11.8)	1 (2.2)	14 (12.1)
Loss of consciousness, n (%)	1 (1.9)	4 (5.2)	0 (0)	7 (6)
Vertigo, n (%)	7 (13.6)	10 (13.1)	2 (4.4)	7 (6)
Lethargy, n (%)	1 (1.9)	2 (2.6)	1 (2.2)	5 (4.3)
Scan for metastases, n (%)	4 (7.5)	0 (0)	4 (9)	12 (10.3)
Other ****, n (%)	12 (22.6)	4 (5.2)	12 (26.6)	10 (8.6)

*Symptoms deemed not associated with the meningioma in question

** p = 0.5

*** p = 0.046

**** Data on “other” available in Supplementary Table 1

### Baseline characteristics

**Fig. 1:**
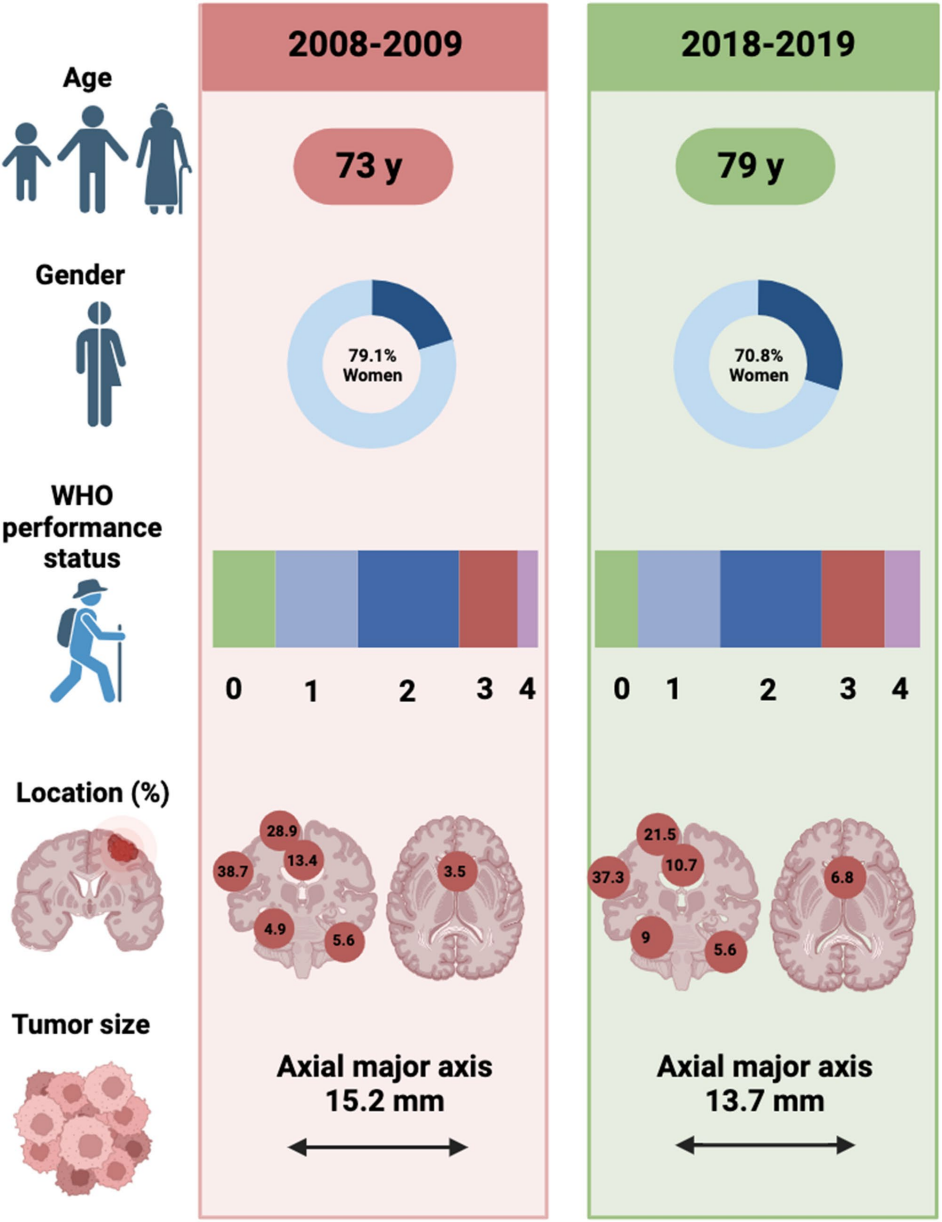
Patient baseline characteristics. Created with BioRender.com

Patient baseline characteristics are shown in Fig. 1. Age at diagnosis was significantly different in the two groups, with patients between years 2008–2009 being younger than patients in 2018–2019 (73 vs. 79 years, *p* = 0.03). There were no significant differences in WHO performance status between the patients identified in 2008–2009 compared to 2018–2019 (*p* = 0.095). On a group level there was no significant difference in the existence of comorbidity among the patients (*p* = 0.07), but when analyzing per comorbidity, there was a significant difference in the occurrence of cancer without metastases identified, which was more common among patients in 2018–2019 (23.6 vs. 11.6%, *p* = 0.009, Table 2). Hypertension was the most common registered comorbidity in both cohorts, followed by diabetes.

**Table 2:** Patient comorbidities at diagnosis

Patient comorbidities	2008–2009	2018–2019	p value
Total number of patients with any comorbidity, n (%)*	99 (76.7)	136 (84.5)	0.07
Hypertension, n (%)	66 (51.2)	88 (54.7)	0.32
Myocardial infarction, n (%)	17 (13.2)	14 (8.7)	0.22
Congestive heart failure, n (%)	13 (10.1)	25 (15.5)	0.17
Peripheral vascular disease, n (%)	10 (7.8)	6 (3.7)	0.14
Stroke/TIA, n (%)	7 (5.4)	6 (3.7)	0.49
Hemi/paraplegia, n (%)	3 (2.3)	4 (2.5)	0.93
Diabetes, n (%)	23 (17.8)	29 (18)	0.97
COPD/Asthma, n (%)	17 (13.2)	26 (16.1)	0.48
Renal disease, n (%)	6 (4.7)	14 (8.7)	0.18
Peptic ulcer disease, n (%)	2 (1.6)	6 (3.7)	0.26
Cancer without metastases, n (%)	15 (11.6)	38 (23.6)	0.009
Cancer with metastases, n (%)	4 (3.1)	12 (7.5)	0.11
Rheumatic disease, n (%)	9 (7)	15 (9.3)	0.47
Depression, n (%)	18 (14)	15 (9.3)	0.22
Dementia, n (%)	7 (5.4)	8 (5.0)	0.86
On warfarin, n (%)	10 (7.3)	6 (3.7)	0.14

*Each individual may be diagnosed with several of the below mentioned comorbidities

### Radiological tumor characteristics

The largest tumor diameter measured in the axial plane was in median 15.2 mm in 2008–2009, and 13.7 mm 2018–2019, (*p* = 0.19, Fig. 1). The three most common tumor locations in both groups were in descending order convexity, parasagittal, and parafalcine. In both groups, most tumors were in the left hemisphere. Neither location nor laterality differed significantly between the groups (Table 3; Fig. 1).

**Table 3:** Radiological tumor characteristics

Radiological tumor characteristics	2008–2009	2018–2019	p value
Number of meningiomas			0.57
Single, n (%)	117 (90.7)	149 (92.5)	
Multiple, n (%)	12 (9.3)	12 (7.5)	
Venous sinus nearby^b^			0.9
Superior sagittal sinus, n (%)	47 (33.1)	53 (29.9)	
Cavernous sinus, n (%)	4 (2.8)	11 (6.2)	
Sigmoid sinus, n (%)	2 (1.4)	3 (1.7)	
Transverse sinus, n (%)	4 (2.8)	4 (2.3)	
In contact with critical neurovascular structures^a^			0.84
Yes, n (%)	5 (3.5)	7 (4)	
No, n (%)	137 (96.5)	170 (96)	
Laterality^a^			0.45
Mainly left sided, n (%)	71 (50)	90 (50.8)	
Mainly right sided, n (%)	55 (38.7)	72 (40.7)	
Midline, n (%)	16 (11.3)	14 (7.9)	

^a^May exceed number of patients due to multiple meningiomas per patient

^b^In contact and/or invasion

## Outcomes

At diagnosis, most patients in both groups were immediately discharged from further follow-up. Active monitoring was performed in 14.7% of patients diagnosed in 2008–2009, and in 18% of patients diagnosed in 2018–2019. Only 1.6% and 0.6%, respectively, were recommended surgery after the diagnostic scan. No patients during either time period received radiosurgery or radiotherapy. On group level, management decisions at diagnosis did not significantly differ between the two cohorts (*p* = 0.6). During the first 3 years after diagnosis, there was a significant difference in the number of performed follow-up scans between groups (*p* = 0.04) as 12.4% respective 11.2% of patients underwent one follow-up scan, but in the 2018–2019 cohort 6.8% underwent two follow-up scans compared to none of patients in the 2008–2009 cohort (Table 4).

**Table 4:** Patient outcome characteristics

Outcome	2008–2009	2018–2019	p value
Management decision among all patients at diagnosis			0.6
Active monitoring, n (%)	19 (14.7)	29 (18)	
Surgery, n (%)	2 (1.6)	1 (0.6)	
Discharge from outpatient care, n (%)	93 (72.1)	114 (70.8)	
Lost to follow up, n (%)	7 (5.4)	10 (6.2)	
Dead, n (%)	8 (6.2)	7 (4.3)	
Number of follow-up scans among patients with initial or resumed* active monitoring within 3 years after diagnosis			0.04
1, n (%)	16 (12.4)	18 (11.2)	
2, n (%)	0 (0)	11 (6.8)	
3, n (%)	2 (1.6)	4 (2.5)	
Management decision at last follow up within 3 years of diagnosis of those under initial or resumed* active monitoring			0.14
Active monitoring, n (%)	6 (31.5)	18 (54.5)	
Surgery, n (%)	2 (10.5)	4 (12.1)	
Discharge from outpatient care, n (%)	6 (31.5)	9 (27.3)	
Lost to follow up, n (%)	5 (26.3)	2 (6.1)	
New meningioma related symptoms among all patients within 3 years after diagnosis, n (%)	2 (1.6)	0 (0)	0.16
Death within first 3 years after diagnosis, n (%)	38 (29.5)	57 (35.4)	0.2

*Total number of patients with follow-up scan may exceed the number of patients initially assigned active monitoring as some patients had their brain rescanned for other causes, where change in meningioma was seen and they were resumed to active monitoring

Within 3-year of follow-up, 1.6% of all patients diagnosed in 2008–2009 experienced new symptoms attributed to the meningioma. No patients in 2018–2019 have yet experienced any new meningioma related symptoms. After a maximum of 3 years of follow-up, there was no significant difference in management decision at last follow-up between the two time periods (*p* = 0.14). However, in the later time period, four patients had been resumed active monitoring after scan of the brain for other cause, where growth had been seen. At the last follow-up within 3 years after diagnosis, patients diagnosed with incidental meningioma in 2008–2009 were most likely to be suggested discharge from outpatient care or continued active monitoring (31.5% respectively). However, among patients diagnosed in 2018–2019, continued active monitoring was the most common approach (54.5%). Within the first 3 years of diagnosis, 29.5 and 35.4% of patients from the respective time periods were deceased (*p* = 0.2) (Table 4).

### Distribution of scans

Within the region of Western Sweden, Sahlgrenska University hospital is the sole university hospital, and it is surrounded by six community hospitals. Primarily three private clinics provide CTs and MRIs of the brain in this region, and the usage of private and community hospital scanners between 2008 and 2018 is presented in supplementary Table 2. In the private sector 2459 CT or MRI of the brain were performed in 2008, and 8716 in 2018, a 254% increase over time. In a pooled analysis of the surrounding community hospitals, the usage increased from 33,691scans in 2008 to 49,555 scans in 2018, an increase of 47%.

## Discussion

This study demonstrates that there was a non-significant increase of patients diagnosed with an incidental meningioma 2018–2019 compared to 2008–2009. Patient age was significantly higher in the later period, and more scans were performed with contrast enhancement. There was a comparable number of brain scans performed between 2008 and 2009 and 2018–2019, with a 120% increase in the usage of contrast enhanced diagnostic scans in the latter period, irrespective of modality, as well as an increased usage of MRI in acute diagnostics.

There was no increase in the total number of patients undergoing CT or MRI in the two study periods, but in 2018–2019 significantly more patients diagnosed with an incidental meningioma was diagnosed using MRI. In a study of imaging trends in England between 2013 and 2017 there was a striking increase in the rate of scans in patients older than 65 years between 2013 and 2017 [18]. It is possible that the difference between our results and the results from the above-mentioned study may depend on an increasing availability of CT and MRI scanners in the private healthcare sector available to the primary health care setting (i.e., GP/family doctors’ offices), something that historically has not been the norm in Sweden. The number of scans performed in the private sector has increased by 250% between 2008 and 2018. However, in the previously mentioned study from England, it was found that most of the increase was due to CT head scans performed in the emergency department, which in Gothenburg is only available at publicly funded hospitals, and there was a very low rate of scans performed due to a family doctor request or from an outpatient setting [18]. Sahlgrenska is a large University Hospital, and the increasing availability of CT and MRI scanners in smaller hospitals with decentralization of some examinations has likely prevented the expected increased rate of brain CT/MRI scans performed at Sahlgrenska. However, as numerically more incidental meningiomas were detected, improvements in image quality and slice-thickness in addition to more contrast enhanced scans may have contributed to the increase in detection rate between 2008 and 2018.

There was no difference between the time periods in the indication for scan, nor patient comorbidity, besides non-metastatic cancer. However, patients in the latter cohort were older. Another observation was that significantly more patients in 2018–2019 with non-metastatic cancer underwent brain scanning in search of brain metastases, especially among elder patients aged above 70 years of age. This could be related to evolving guidelines regarding the management of brain metastases [19], as the possibilities for treatment/cure of brain metastases has improved over the last decade [20]. Thus, one might speculate that with better treatment options one might be more prone to scanning a patient with already known cancer for metastases around the body, including the brain. It is also possible that patients with such complicated comorbidity would be more likely to be referred for brain scanning at the University Hospital where they are likely also undergoing treatment, whilst healthier and young patients be referred either to a community hospital or the private sector. This shift in demography may have contributed to our findings.

No differences were seen in tumor size, location, or management decision at diagnosis between 2008 and 2009 and 2018–2019. Within the first 3 years after diagnosis however, significantly more patients in 2018–2019 had undergone two scans of active monitoring as compared to patients in 2008–2009. Incidental findings can cause economic issues, and in many cases, there is no clear patient-centered benefit to their identification [21]. Out of 5800 studied participants in a population-based cohort with median age 65 years, 143 meningiomas were identified (2,5%). Close to 65% of the meningiomas in this group were referred for further assessment, but only 10% of them underwent intervention [22], in total one intervention per 644 performed scans. In our sample, the corresponding number is one intervention per 5000 performed scans. This cost money, leads to additional workload, and might cause anxiety for the patient; hence the risk-benefit balance is not entirely clear. This offset has also been studied for incidental intracranial aneurysms, where despite the potential severity in the event of a subarachnoid hemorrhage and the existence of an effective treatment, empiric screening was not found to be cost effective even in high risk populations [23, 24].

A Choosing Wisely campaign from 2014 strongly recommended against the radiological scanning of asymptomatic patients after syncope, head-trauma, and a normal neurological examination [25]. In our cohort head injury was among the most common indication for imaging. For patients with obvious sign of traumatic brain injury, or presenting with other “red flags”, neuroimaging has obvious benefits, such as guiding medical and neurosurgical interventions [26]. However, for patients without symptoms, other factors must be considered, such as increased length of hospital stay, harm and cost from incidental findings, and risk of subsequent cancer due to exposure to ionizing radiation [27, 28]. Meningiomas presenting with focal neurological deficits and seizures have clear management guidelines, with surgery or radiotherapy as first line treatment [29], but that is not the case for incidental and asymptomatic meningioma. European Association of Neuro-Oncology and Response assessment in Neuro-Oncology guidelines suggest active monitoring, but this seems unreasonable in many of the scenarios where incidental meningiomas are detected [29, 30]. The guidelines further state that treatment should be kept for situations where symptoms develop, substantial growth occurs, or there is danger to sensitive areas [29]. In lack of better selection criteria, clinical judgement is needed with respect to how incidental meningiomas are managed upon detection. Of note in the results of this study is that during both time periods around 30% of patients were deceased within 3 years after diagnosis, due to causes other than their meningioma. When discussing potential treatment and follow-up with a patient with newly diagnosed incidental meningioma, it is therefore of importance to consider not only factors related to the meningioma itself, but also factors related to the entire patient. Even at the event of growth, or even mild symptom development, one might consider withholding treatment or follow-up in patients with significant comorbidity, or short life expectancy.

### Limitations

There are several limitations to this study. The data is primarily limited to Sahlgrenska University Hospital, leaving the possibility for bias from excluding examinations performed by smaller hospitals in the region and examinations performed by the private sector. However, this should not obviously affect examinations performed in the emergency department setting. We did not have access to data on image quality, such as slice thickness or the Tesla (T) of used MRI-scanners, which could have an impact on the detection rate of incidental findings. Among the patients identified with an incidental meningioma, many underwent only a CT scan as part of their diagnostic workup. It was not possible to draw conclusions regarding if MRI increased the detection rate, as too few patients underwent MRI. The follow up time between the two time periods differ to such an extent that it is difficult at this time point to draw specific conclusions concerning long-term differences in management and outcome.

## Conclusions

Numerically more patients were diagnosed with an incidental asymptomatic meningioma between 2018 and 2019 compared to 2008–2009, however not statistically significant, even though the number of scans performed each year was the same. This could perhaps be attributed improved image quality, more use of contrast enhanced scans as well as a demographic shift as more patients were older, and more often had non-metastatic cancer. In the latter period, search for brain metastases was more common. There was no significant difference in the rate of surgical management decision at diagnosis, but within 3 years of follow up significantly more patients in the 2018–2019 cohort had been followed with active monitoring. After 3 years of follow-up, a substantial number of patients from both times periods were deceased due to causes unrelated to their meningioma, which should be taken into account when considering regimes for treatment or follow-up of incidental meningiomas,

### Supplementary Information

Below is the link to the electronic supplementary material.

Supplementary material 1 (DOCX 42.8 kb)

Supplementary material 2 (DOCX 16.0 kb)

Supplementary material 3 (DOCX 13.9 kb)
