# Supplementary material for: Incidence, management, and outcome of incidental meningioma: what has happened in 10 years?
Source: J Neurooncol. 2023 Nov 8;165(2):291–9. doi: 10.1007/s11060-023-04482-5 (PMC10689551; doi:10.1007/s11060-023-04482-5)
Supplement: Supplementary file 1 — Supplementary material 1 (DOCX 42.8 kb) [file 11060_2023_4482_MOESM1_ESM.docx]

***Supplementary figure 1****: Resulting flow-chart of search strategy 2008-2009 and 2018-2019*

CT or MRI of brain performed in 2018-2019
n = 35 841

CT or MRI of brain performed in 2008-2009
n = 31 166

**Identification**

Duplicates or missing personal ID-number

n = 13 854

Duplicates or missing personal ID-number

n = 8 907

Number of unique patients undergoing CT or MRI of brain in 2018-2019

n = 21 987

Number of unique patients undergoing CT or MRI of brain in 2008-2009

n = 22 259

**Screening**

Patients excluded due to:

Previous diagnosis= 867

Symptoms at diagnosis=88

No clinical data available=289

Not meningioma=82

Normal scan=14
Other=77

Patients excluded due to:

Previous diagnosis= 959

Symptoms at diagnosis=64

No clinical data available=298

Not meningioma=46

Normal scan=14

Other=92

Number of patients keyword “meningioma” in radiological report in 2018-2019

n = 1 634

Number of patients keyword “meningioma” in radiological report in 2008-2009

n = 1 546

**Eligibility**

Patients with incidental meningioma included in 2018-2019
n = 161

Patients with incidental meningioma included in 2008-2009
n = 129

**Included**
