# Supplementary material for: Incidence, management, and outcome of incidental meningioma: what has happened in 10 years?
Source: J Neurooncol. 2023 Nov 8;165(2):291–9. doi: 10.1007/s11060-023-04482-5 (PMC10689551; doi:10.1007/s11060-023-04482-5)
Supplement: Supplementary file 2 — Supplementary material 2 (DOCX 16.0 kb) [file 11060_2023_4482_MOESM2_ESM.docx]

***Supplementary table 1****: Other indication for diagnostic scan*

|  | 2008-2009 | | 2018-2019 | |
| --- | --- | --- | --- | --- |
| Indication for scan* | **Age < 70** | **Age ≥ 70** | **Age <70** | **Age ≥70** |
| Pain behind eye, n (%) | 1 (1.9) | 0 (0) | 1 (2.2) | 0 (0) |
| Motor symptoms, n (%) | 1 (1.9) | 0 (0) | 2 | 0 (0) |
| Cerebral SLE, n (%) | 1 (1.9) | 0 (0) | 0 (0) | 0 (0) |
| Control scan after surgery, n (%) | 2 (3.8) | 0 (0) | 0 (0) | 0 (0) |
| Normal pressure hydrocephalus, n (%) | 1 (1.9) | 0 (0) | 0 (0) | 1 (0.9) |
| Start of chemotherapy, n (%) | 1 (1.9) | 0 (0) | 0 (0) | 0 (0) |
| Control after stroke, n (%) | 0 (0) | 1 (1.3) | 0 (0) | 0 (0) |
| Control before carotid surgery, n (%) | 0 (0) | 1 (1.3) | 0 (0) | 0 (0) |
| Research, n (%) | 1 (1.9) | 0 (0) | 1 (2.2) | 0 (0) |
| Sensory symptoms, n (%) | 2 (3.8) | 0 (0) | 1 (2.2) | 1 (0.9) |
| Infection, n (%) | 0 (0) | 2 (2.6) | 0 (0) | 1 (0.9) |
| Pain in ear, n (%) | 1 (1.9) | 0 (0) | 0 (0) | 0 (0) |
| Knife stab to the throat, n (%) | 1 (1.9) | 0 (0) | 0 (0) | 0 (0) |
| Seizure, n (%) | 0 (0) | 0 (0) | 2 (4.4) | 2 (1.7) |
| Primary brain tumor, n (%) | 0 (0) | 0 (0) | 2 (4.4) | 2 (1.7) |
| Syncope, n (%) | 0 (0) | 0 (0) | 1 (2.2) | 1 (0.9) |
| Cardiac arrest, n (%) | 0 (0) | 0 (0) | 0 (0) | 1 (0.9) |
| Carnes syndrome, n (%) | 0 (0) | 0 (0) | 1 (2.2) | 0 (0) |
| MEN1, n (%) | 0 (0) | 0 (0) | 1 (2.2) | 0 (0) |
| Multiple sclerosis, n (%) | 0 (0) | 0 (0) | 0 (0) | 1 (0.9) |

*Symptoms deemed not associated with the meningioma in question
