# Supplementary material for: Incidence, management, and outcome of incidental meningioma: what has happened in 10 years?
Source: J Neurooncol. 2023 Nov 8;165(2):291–9. doi: 10.1007/s11060-023-04482-5 (PMC10689551; doi:10.1007/s11060-023-04482-5)
Supplement: Supplementary file 3 — Supplementary material 3 (DOCX 13.9 kb) [file 11060_2023_4482_MOESM3_ESM.docx]

***Supplementary table 2****: Evolution of usage of private sector and community hospital scanners for CT and MRI of the brain between 2008-2018*

|  | 2008 | 2009 | 2010 | 2011 | 2012 | 2013 | 2014 | 2015 | 2016 | 2017 | 2018 |
| --- | --- | --- | --- | --- | --- | --- | --- | --- | --- | --- | --- |
| Private sector (n) | 2459 | 3042 | 3322 | 3998 | 4348 | 5525 | 6741 | 7835 | 8669 | 7292 | 8716 |
| Community hospital (n) | 33691 | 37452 | 36827 | 43061 | 40649 | 42916 | 43176 | 46496 | 44824 | 46397 | 49555 |
